# Severe acute respiratory coronavirus virus 2 (SARS-CoV-2) infections occurring in healthcare workers after booster vaccination: A comparison of delta versus omicron variants

**DOI:** 10.1017/ash.2022.239

**Published:** 2022-08-18

**Authors:** Kimberly D. Reeves, Christopher M. Polk, Laura A. Cox, Robert T. Fairman, Gregory A. Hawkins, Catherine L. Passaretti, Mindy M. Sampson

**Affiliations:** 1 Center for Precision Medicine, Wake Forest School of Medicine, Winston-Salem, North Carolia; 2 Department of Internal Medicine, Wake Forest School of Medicine, Winston-Salem, North Carolina; 3 Division of Infection Diseases, Department of Medicine, Atrium Health, Charlotte, North Carolina; 4 Department of Health Policy & Behavioral Sciences, School of Public Health, Georgia State University, Atlanta, Georgia; 5 Department of Biochemistry, Wake Forest School of Medicine, Winston-Salem, North Carolina

## Abstract

In this study, we used genomic sequencing to identify variants of severe acute respiratory coronavirus virus 2 (SARS-CoV-2) in healthcare workers with coronavirus disease 2019 (COVID-19) after receiving a booster vaccination. We compared symptoms, comorbidities, exposure risks, and vaccine history between the variants. Postbooster COVID-19 cases increased as the SARS-CoV-2 omicron variant predominated.

During the coronavirus disease 2019 (COVID-19) pandemic, multiple variants of severe acute respiratory coronavirus virus 2 (SARS-CoV-2) have emerged. Mutations in the viral genome have developed, conveying improvements in viral fitness and allowing greater infectivity and predominance of certain variants during different waves of the pandemic.^1^ During the summer of 2021, the SARS-CoV-2 δ (delta) variant predominated in the United States, but currently, most COVID-19 cases in the United States are due to the SARS-CoV-2 ο (omicron) variant.^2^

Prior COVID-19 vaccination is highly protective against severe COVID-19 disease for immune-competent individuals, although that protection may wane with time since vaccination.^3^ The degree of protection that COVID-19 vaccines provide may differ between variants. Mutations in the SARS-CoV-2 spike-protein region of the SARS-CoV-2 omicron virus may help it escape the antibody response generated by the vaccines, potentially leading to less protection from the SARS-CoV-2 omicron variant.^4,5^ Booster vaccinations are recommended to prolong immune protection from infection and to bolster immune defenses against variants such as the SARS-CoV-2 omicron variant.^6,7^ Booster vaccination reduces rates of COVID-19 compared to thowse who are unvaccinated or those who received 2 doses. However, data suggest a higher likelihood of becoming infected with the SARS-CoV-2 ο (omicron) variant than the SARS-CoV-2 delta variant, even after a booster vaccine.^8,9^ We examined COVID-19 cases in healthcare workers (HCWs) following booster vaccinations in a large healthcare system to determine the rates of postbooster vaccination COVID-19 cases caused by the SARS-CoV-2 delta and omicron variants.

## Methods

With the approval of our institutional review board, we conducted a retrospective cohort study of 6,586 HCWs who tested positive for SARS-CoV-2 from October 10, 2021, to January 21, 2022. SARS-CoV-2 polymerase chain reaction (PCR) testing of nasopharyngeal swab samples was performed on various platforms, including rapid home antigen tests. Only HCWs who had a positive reverse transcription (RT)-PCR Roche Cobas test (Roche Diagnostics, Indianapolis, IN) were included for genomic sequencing. SARS-CoV-2 genomic sequencing was performed on randomly selected samples with cycle threshold (Ct) values <30. Briefly, total RNA was isolated from each sample and sequenced using the MiSeq, NextSeq 500, or NovaSeq 6000 platform (Illumina, San Diego, CA). Sequence reads were mapped to the SARS-CoV-2 reference (NC_045512), and the consensus genome sequence for each sample was imported into GISAID (https://www.gisaid.org) for lineage designation.

HCW symptoms and vaccination history were assessed using a standardized data collection tool. A postbooster infection was defined as a positive test that occurred ≥14 days after receiving a COVID-19 booster vaccination. Obesity was defined as having a body mass index ≥30. High-risk exposure was defined as traveling without social distancing in the previous 14 days or attending a large group gathering of >25 people outside or >10 inside. Differences in symptoms, medical history, and sequencing results were analyzed using the χ^2^ test. When cell counts were <5, the Fisher exact test was utilized. To assess differences in means, Student *t* tests were utilized, assuming equal variances. All analyses were conducted using SAS version 9.4 software (SAS Institute, Cary, NC). Listwise deletion was for complete case analysis. For all analyses, α = 0.05.

## Results

**Figure 1.**
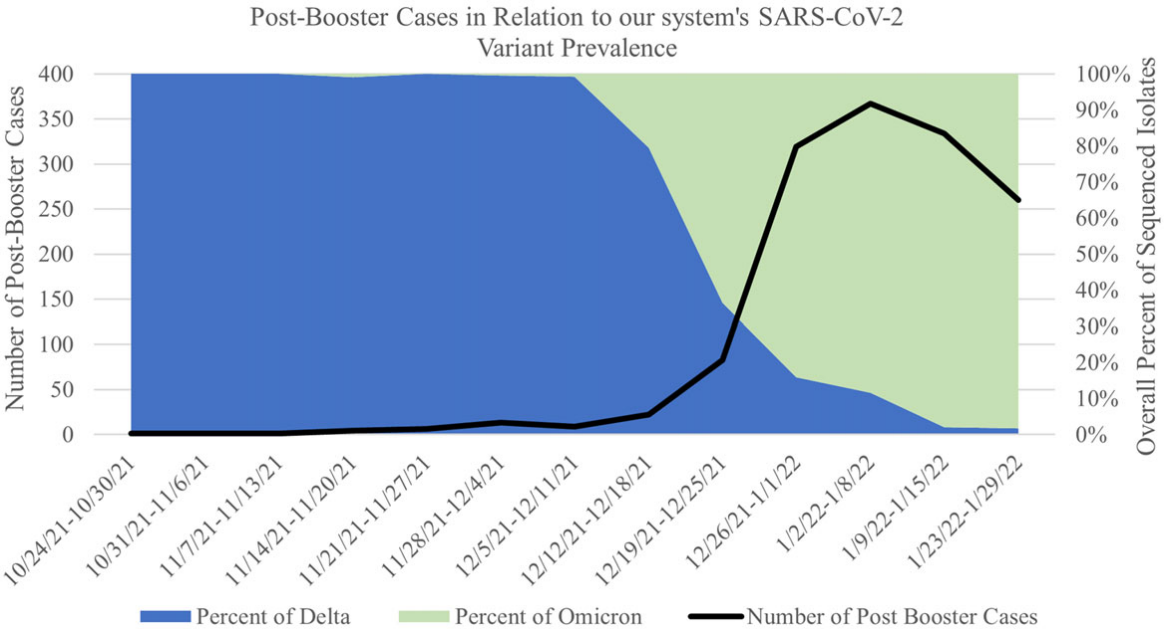
Number of post-booster cases in relation to the percentage of delta and omicron variants over time.

Among the 6,586 HCWs, 1,844 had postbooster infections. HCW postbooster infections increased between October and January, peaking in January and corresponding with dominance of the SARS-CoV-2 omicron variant in sequenced samples (Fig. 1). The SARS-CoV-2 omicron variant became the predominant variant in our area after December 21, 2021. Prior to that, we had only 70 postbooster infections. Prior to December 21, we performed genomic sequencing on 29 (41%) of the postbooster COVID-19 cases; after December 21, we performed genomic sequencing on 290 (16%) of the postbooster COVID-19 cases. Moreover, 279 HCWs had both symptom history and genomic sequencing results, which were included in the analysis. Demographic and medical history did not differ between HCWs infected with the SARS-CoV-2 delta or omicron variant (Table 1). Although we detected no statistically significant differences in race or ethnicity of those with COVID-19 caused by the SARS-CoV-2 delta and omicron variants, 18.5% of those with the SARS-CoV-2 omicron variant reported being Black or African American, but none reported a case with the SARS-CoV-2 delta variant.

**Table 1.** Descriptive Statistics for Healthcare Workers, Stratified by WHO Lineage, (N=279)

Variable	Lineage		*P* Value
	SARS-CoV-2 Delta Variant, No. (%)	SARS-CoV-2 Omicron Variant, No. (%)	
**Demographics**			
Age, mean y (SD)	43.32 (11.14)	40.53 (11.23)	.2984
**Race**			.1324
American Indian	0 (0.0)	1 (0.39)	
Asian	2 (10.53)	13 (5.12)	
Black or African American	0 (0.0)	47 (18.50)	
Middle Eastern or North African	0 (0.0)	0(0.0)	
Native Hawaiian	0 (0.0)	1 (0.39)	
White or Caucasian	15 (78.95)	179 (70.47)	
Other	2 (10.53)	13 (5.12)	
**Ethnicity**			.5747
Hispanic or Latinx	0(0.0)	13 (5.12)	
Not Hispanic or Latinx	18 (94.74)	234 (92.13)	
Other/Not Specified	1 (5.26)	7 (2.76)	
**Sex**			1
Female	16 (84.21)	207 (81.50)	
Male	3 (15.79)	47 (18.50)	
**Chills**			.6149
No	19 (100.00)	139 (92.67)	
Yes	0 (0.0)	11 (7.33)	
**Fever**			.784
No	15 (78.95)	11 (74.0)	
Yes	4 (21.05)	39 (26.00)	
**Myalgia**			.7384
No	10 (52.63)	85 (56.67)	
Yes	9 (47.37)	65 (43.33)	
**Headache**			.6289
No	11 (57.89)	76 (50.67)	
Yes	8 (42.11)	74 (49.33)	
**Sore Throat**			.2131
No	12 (63.16)	72 (48.00)	
Yes	7 (36.84)	78 (52.00)	
**Nausea/Vomiting**			1
No	16 (84.21)	127 (84.67)	
Yes	3 (15.79)	23 (15.33)	
**Diarrhea**			1
No	17 (89.47)	128 (85.33)	
Yes	2 (10.53)	22 (14.67)	
**Fatigue**			.8084
No	10 (52.63)	85 (56.67)	
Yes	9 (47.37)	65 (43.33)	
**Congestion**			1
No	3 (15.79)	30 (20.00)	
Yes	16 (84.21)	120 (80.00)	
**Cough**			**.0129**
No	10 (52.63)	38 (25.33)	
Yes	9 (47.37)	112 (74.67)	
**Shortness of breath**			0.6993
No	18 (94.74)	132 (88.00)	
Yes	1 (5.26)	18 (12.00)	
**Difficulty breathing**			1
No	19 (100.00)	150 (0.0)	
Yes	0 (0.0)	0 (0.0)	
New loss of taste and/or smell			**.0107**
No	13 (68.42)	134 (89.33)	
Yes	6 (31.58)	16 (10.67)	
**Pregnant**			.0949
No	18 (94.74)	173 (68.11)	
Yes	0	7 (2.76)	
Unknown	1 (5.26)	74 (29.13)	
**Obese**			.3146
No	15 (78.95)	167 (65.75)	
Yes	4 (21.05)	87 (34.25)	
Unknown	0	0	
**Type 1 Diabetes**			1
No	19 (100.00)	244 (29.06)	
Yes	0	0	
Unknown	0	10 (3.94)	
**Type 2 Diabetes**			1
No	19 (100.00)	234 (92.13)	
Yes	0	8 (3.15)	
Unknown	0	12 (4.75)	
**Chronic Kidney Disease**			1
No	19 (100.00)	243 (95.67)	
Yes	0	0	
Unknown	0 (0)	11 (4.33)	
**End-stage kidney disease**			1
No	19 (100.00)	243 (95.67)	
Yes	0	1 (0.39)	
Unknown	0	10 (3.94)	
**Chronic liver disease**			1
No	19 (100.00)	243 (95.67)	
Yes	0	2 (0.79)	
Unknown	0	9 (3.54)	
**Hypertension**			.5162
No	18 (94.74)	208 (81.89)	
Yes	1 (5.26)	33 (12.99)	
Unknown	0	13 (5.12)	
**Coronary artery disease**			.2492
No	18 (94.74)	224 (88.19)	
Yes	1 (5.26)	6 (2.36)	
Unknown	0	24 (9.45)	
**Other heart disease**			.8361
No	18 (94.74)	217 (85.43)	
Yes	0	8 (3.15)	
Unknown	1 (5.26)	29 (11.42)	
**COPD**			.9049
No	18 (97.74)	216 (85.04)	
Yes	1 (5.26)	16 (6.30)	
Unknown	0	22 (8.66)	
**Current smoker**			1
No	19 (100)	236 (92.91)	
Yes	0	10 (3.94)	
Unknown	0	8 (3.15)	
**Past smoker**			.3416
No	14 (73.68)	211 (83.07)	
Yes	4 (21.05)	34 (13.39)	
Unknown	1 (5.26)	9 (3.54)	
**Current vaper**			.7106
No	19 (100)	234 (92.13)	
Yes	0 (0)	4 (1.57)	
Unknown	0	16 (6.30)	
**Past vaper**			1
No	18 (94.74)	236 (92.91)	
Yes	0 (0)	3 (1.18)	
Unknown	1 (5.26)	15 (5.91)	
**Job category**			.7075
Doctor, advance practitioner	4 (21.05)	47 (18.50)	
Nurse	8 (42.11)	84 (33.07)	
Healthcare technician^a^	2 (10.53)	26 (10.24)	
Other	5 (26.32)	97 (38.19)	
**High-risk exposure**			.4842
No	18 (94.74)	221 (87.01)	
Yes	1 (5.26)	33 (12.99)	
**Clear exposure**			1
No clear community exposure	8 (42.11)	98 (35.58)	
Yes, household contact is positive	7 (36.84)	81 (31.89)	
All other known contacts	4 (21.05)	71 (27.95)	
**Prior COVID-19**			.6302
No	17 (89.47)	237 (93.31)	
Yes	2 (10.53)	17 (6.69)	
**Vaccine dose 1**			.0661
JNJ-78436735	2 (10.53)	2 (0.79)	
mRNA-1273	0	5 (1.97)	
BNT162b2	17 (89.47)	246 (96.85)	
Unknown	0	1 (.39)	
**Vaccine dose 2**			**.0111**
JNJ-78436735	0	0	
mRNA-1273	0	5 (1.97)	
BNT162b2	16 (84.21)	246 (96.85)	
Unknown	3 (15.79)	3 (1.18)	
**Booster product**			**.0252**
JNJ-78436735	0	0	
mRNA-1273	3 (15.79)	7 (2.76)	
BNT162b2	16 (84.21)	247 (97.24)	
Unknown	0	0	
Days since vaccination series, mean (SD)	292.3 (32.05)	325.3 (25.15)	**<.0001**
Days since booster, mean (SD)	57.89 (28.67)	84.36 (28.56)	**<.0001**
Ct value *Orf1*, mean (SD)	21.92 (5.32)	22.01 (3.24)	.9208
Ct value *E*, mean (SD)	22.39 (5.79)	22.20 (3.26)	.8402
**Hospitalized**			.3574
No	17 (89.47)	239 (94.09)	
Yes	0	1 (.39)	
Unknown	2 (10.53)	14 (5.51)	

Note. SD, standard deviation, COPD, chronic obstructive pulmonary disease; Ct, cycle. The χ^2^ test was used for cell counts >5, otherwise the Fisher exact test is reported. *T* test was used for means, assuming equal variances.

a Includes certified medical assistants (CMAs), healthcare technicians (HCTs), those with a professional service agreements (PSAs), registered medical assistants (RMAs).

The HCWs in our cohort reported similar COVID-19 symptomology between the SARS-CoV-2 omicron and delta variants (see Table 1). More individuals reported cough with COVID-19 caused by the SARS-CoV-2 omicron variant versus the delta variant (75% vs 47%; χ^2^ = 21.6799; *P* = .0129). Loss of taste or smell was more commonly reported with COVID-19 cases caused by the SARS-CoV-2 delta variant (31% vs 10% omicron variant; χ^2^ = 21.1501; *P* = .0107). There were no statistically significant differences in reporting other COVID-19 symptoms. Only one hospitalization occurred in an individual with COVID-19 caused by the SARS-CoV-2 omicron variant, and no mortalities occurred in either group. Most HCWs with a postbooster infection worked in a patient-facing role. When comparing HCWs who had had SARS-CoV-2 exposure via a household contact, a nonhousehold exposure, and no known contact, we did not detect a statistically significant difference between the SARS-CoV-2 variants.

Most postbooster COVID-19 cases occurred in individuals who received both the BNT162b2 primary vaccination series and the BNT162b2 booster. However, >90% of the vaccinated individuals in our healthcare system received the BNT162b2 booster. Nearly 97% of those with COVID-19 caused by the SARS-CoV-2 omicron variant reported a second dose of BNT162b2 rather than another vaccine, compared to 84.21% of those with with COVID-19 caused by the SARS-CoV-2 delta variant (χ^2^ = 22.4661; *P* = .0111). Of those infected with the SARS-CoV-2 omicron variant, 97% also reported receiving the BNT162b2 booster, compared to 84.21% of those with the delta variant (χ^2^ = 22.4661; *P* = .0252). HCWs infected with the SARS-CoV-2 omicron variant compared to the delta variant had a greater number of days since completion of primary vaccine series [t(186) = −4.75; *P* < .0001] and a greater number of days since booster [t(271) = −3.89; *P* = .0001].

## Discussion

As the SARS-CoV-2 ο (omicron) variant became the predominant variant in our community, we saw more postbooster SARS-CoV-2 infections in HCWs. This increase may be related to the increase in total number of COVID-19 cases and, therefore, potential exposure events. However, postbooster infections became much more common after the SARS-CoV-2 omicron variant predominated and with time since booster vaccination. COVID-19 vaccine efficacy decreased with the SARS-CoV-2 omicron variant, including postbooster efficacy.^10^ The significant increase in postbooster COVID-19 case rates argues for greater immune escape with the SARS-CoV-2 omicron variant and lower vaccine efficacy in our HCW population against the omicron variant. but the design of our study did not allow us to estimate vaccine efficacy between variants.

Despite the increased number of cases, overall illness was mild in our boosted HCWs. Interestingly, the presence or absence of a known SARS-CoV-2 exposure did not differ between postbooster cases when comparing the SARS-CoV-2 omicron and delta variants. Thus, healthcare institutions should continue to encourage HCWs to get tested if symptoms associated with COVID-19 develop, regardless of known exposure.

This study had several limitations. Some data were self-reported, which led to missing or incomplete data. Additionally, to accommodate the tracking of HCWs during the COVID-19 surge cause by the SARS-CoV-2 omicron variant, our survey instrument changed. This resulted in the elimination of symptom assessment after January 16, 2022.

As the COVID-19 pandemic transitions to an endemic infection status, most cases will be expected to occur in individuals with some prior immunity, including postbooster infections. Our data suggest that HCWs continue to be affected as new SARS-CoV-2 variants and COVID-19 surges occur, despite being a highly immunized population. Although most infections will be mild in a highly vaccinated workforce, our study reinforces the importance of vigilance in protective measures like the use of masks during periods of higher community transmission, symptom screening, and liberal sick leave policies to avoid exposure to more susceptible patients. Whether more frequent booster vaccinations would overcome the waning efficacy of COVID-19 vaccination in the SARS-CoV-2 omicron and future SARS-CoV-2 variants is uncertain. Ongoing vigilance for viral surveillance and mitigation strategies in HCWs to prevent nosocomial infections, especially during periods of high viral prevalence, will be needed.
